# No Impact of Cerebellar Anodal Transcranial Direct Current Stimulation at Three Different Timings on Motor Learning in a Sequential Finger-Tapping Task

**DOI:** 10.3389/fnhum.2021.631517

**Published:** 2021-02-05

**Authors:** Carine Nguemeni, Annika Stiehl, Shawn Hiew, Daniel Zeller

**Affiliations:** Department of Neurology, University Hospital of Würzburg, Würzburg, Germany

**Keywords:** cerebellar tDCS, finger-tapping task, timing, motor learning, task retention

## Abstract

**Background**: Recently, attention has grown toward cerebellar neuromodulation in motor learning using transcranial direct current stimulation (tDCS). An important point of discussion regarding this modulation is the optimal timing of tDCS, as this parameter could significantly influence the outcome. Hence, this study aimed to investigate the effects of the timing of cerebellar anodal tDCS (ca-tDCS) on motor learning using a sequential finger-tapping task (FTT).

**Methods**: One hundred and twenty two healthy young, right-handed subjects (96 females) were randomized into four groups (During_sham_, Before, During_real_, After). They performed 2 days of FTT with their non-dominant hand on a custom keyboard. The task consisted of 40 s of typing followed by 20 s rest. Each participant received ca-tDCS (2 mA, sponge electrodes of 25 cm^2^, 20 min) at the appropriate timing and performed 20 trials on the first day (T1, 20 min). On the following day, only 10 trials of FTT were performed without tDCS (T2, 10 min). Motor skill performance and retention were assessed.

**Results**: All participants showed a time-dependent increase in learning. Motor performance was not different between groups at the end of T1 (*p* = 0.59). ca-tDCS did not facilitate the retention of the motor skill in the FTT at T2 (*p* = 0.27). Thus, our findings indicate an absence of the effect of ca-tDCS on motor performance or retention of the FTT independently from the timing of stimulation.

**Conclusion**: The present results suggest that the outcome of ca-tDCS is highly dependent on the task and stimulation parameters. Future studies need to establish a clear basis for the successful and reproducible clinical application of ca-tDCS.

## Introduction

Transcranial direct current stimulation (tDCS) is a non-invasive brain stimulation technique that has impressively grown in popularity in clinical research within the last two decades (Stagg et al., 2018). It has been widely used as a simple and safe method to modulate cortical excitability of the human brain both in the healthy and in the pathological context (Dedoncker et al., 2016; Angius et al., 2019; de Moura et al., 2019).

While most studies focus on the influence of tDCS on cortical areas, the interest in cerebellar transcranial direct current stimulation (ctDCS) has grown rather recently, mainly after Galea et al. (2009) described polarity-depended excitability changes using ctDCS. Twenty-five minutes of anodal stimulation increased the connectivity between the cerebellum and the primary motor cortex (M1), whereas connectivity was decreased by cathodal stimulation (Galea et al., 2009). An additional number of recent studies have shown that tDCS induces significant changes in cerebellar excitability (Ferrucci et al., 2015).

The cerebellum plays a critical role in motor learning, movement coordination, motor adaptation, and cognitive processing (Thach, 1998; Morton and Bastian, 2006; Buckner, 2013). Indeed, the connections between the cerebellum and the motor cortex are essential for the performance of daily life activities. This is because the cerebellum is closely connected to the motor cortex *via* multiple closed-loop circuits which optimize motor control and motor learning by refining motor inhibition (Wolpert et al., 2011). This represents a key feature of cerebrocerebellar interactions (Kelly and Strick, 2003). In this sense, cerebellar stimulation may functionally affect cerebrocerebellar interactions and modulate functions residing in the motor cortex and the whole brain (Priori et al., 2014). This also explains why any perturbation of the integrity of these interactions results in motor disorders including ataxia, dysmetria, dystonia (Manto et al., 2013).

Therefore, developing non-invasive strategies to modulate cerebellar excitability constitutes an interesting opportunity to further understand the cerebellar function and the potential benefit of cerebellar stimulation for patients with neurological disorders. Regarding motor learning, future applications could include improvement of fine motor functions (Priori et al., 2014) or improving balance control (Manto et al., 2013; Poortvliet et al., 2018), e.g., in stroke patients (Wiestler et al., 2011).

There are, however, several challenges that need to be overcome to use ctDCS successfully in research as well as in clinical settings. The human cerebellar cortex presents a complex architecture in a sheet-like structure and accordion-like folds containing layers of excitatory and inhibitory cells (Motolese et al., 2013). Applying stimulation in a precise direction parallel to the somatodendritic axis of the target cells, which was shown to generate the maximum effect on polarization (Bikson et al., 2004), is therefore not easy to accomplish. Also, inter-individual differences regarding skull thickness and cerebellar architecture limit the finding of congruent effects (Motolese et al., 2013; Guell and Schmahmann, 2020); even when study designs and paradigms are very similar (Rahman et al., 2014; Jalali et al., 2017). Thus, the results obtained from tDCS applied to the cerebrum cannot be simply transferred to the cerebellum (Motolese et al., 2013; van Dun et al., 2016). Furthermore, there is a critical lack of consensus about the application of tDCS (Rezaee and Dutta, 2019).

Therefore, a systematic investigation of the effects of different ctDCS protocols is needed (Ferrucci et al., 2015). This will help to generate maximum effects in further studies and to make ctDCS studies more comparable. Regarding ctDCS protocols, the effects of five main factors need to be investigated: the duration of stimulation, the current intensity, electrode size and placement, the number of ctDCS sessions, and the timing of stimulation (Stagg et al., 2011). In the present study, we focused on the timing of stimulation.

When referring to stimulation timing, one usually distinguishes between offline stimulation and online stimulation. Whereas in online stimulation participants perform a task during the stimulation, offline stimulation can be administered either before or after training a task. Many studies use one of the timing options without justifying their choice in detail (Thair et al., 2017). Not rarely, online stimulation is preferred and justified by simple reasons including: (1) a tight schedule; or (2) the assumption that both online and offline protocols induce the same polarity-specific effects (Schlerf et al., 2015). However, the timing of stimulation concerning the execution of a specific behavioral task might play a pivotal role in the overall outcome of tDCS. In opposition to reports from the stimulation of the motor cortex (Sriraman et al., 2014; Stagg et al., 2018), no study has yet investigated the effect of ctDCS timing on motor performance and retention in motor learning, comparing offline and online stimulation within the same study. Here, we sought to explore the impact of anodal ctDCS at different times (before, during, or after a task) on motor performance and retention using a sequential finger-tapping task (FTT). We hypothesized that anodal ctDCS applied during the task will improve task performance while anodal ctDCS applied after the task will improve consolidation and retention when compared to sham tDCS. A previous study demonstrated that a single session of tDCS to the prefrontal cortex before an implicit sequence learning task does not affect the performance on the task (Savic et al., 2017). As such, we also hypothesized that anodal ctDCS applied before the FTT will neither influence motor performance nor consolidation and will produce results similar to sham tDCS.

The FTT represents a widely used procedural memory task to assess motor skill learning (Walker et al., 2002; Rasch et al., 2009). The cerebellum has been shown to play an important role in the correct execution of this type of motor sequence learning task (Penhune and Steele, 2012; Shimizu et al., 2017). Moreover, patients with cerebellar degeneration fail to show improvement in procedural learning (Pascual-Leone et al., 1993; Gómez-Beldarrain et al., 1998; Shin and Ivry, 2003).

Regarding the ideal cerebellar region to stimulate when modulating FTT performance, studies have analyzed the cerebellar-motor cortex connectivity governing upper-limb motor skill. The individual finger-specific activation shows only weak somatotopic organization with ipsilateral patterns of activation in the lobule V and VIII of the cerebellar hemisphere (Wiestler et al., 2011). Upper-limb representation is also more widely distributed touching face and mouth representation in the superior posterior cerebellum (lobule VI) and lower limb representations in the inferior posterior cerebellum (lobules VIIb-IX; Motolese et al., 2013). Data-driven from cerebellar functional magnetic resonance imaging indicated that the lobules I-VI and lobule VIII of the cerebellar hemisphere are activated during motor processing like moving a finger (Guell and Schmahmann, 2020). Altogether, these data hint at the stimulation of the posterior cerebellum as a good target to evaluate ctDCS on FTT. We chose this region as our target using an electrode montage (Galea et al., 2009) which can effectively affect the lobules VIIb, VIII, and IX in the ipsilateral cerebellum (Rezaee and Dutta, 2019).

Here, we specifically focused our evaluation on the timing of anodal stimulation in one session because multiple studies have suggested a facilitatory effect of the anodal polarity on motor learning with ctDCS (Galea et al., 2011; Jayaram et al., 2012; Hardwick and Celnik, 2014; Herzfeld et al., 2014; Priori et al., 2014; Cantarero et al., 2015). More, a single stimulation of the cerebellum has been shown to lead to significant retention of fine motor skills (Wessel et al., 2016).

## Materials and Methods

### Participants

One hundred and twenty two healthy, right-handed young adults (96 females, 26 males, age range: 19–27 years, mean age: 22.5 ± 2.2) were recruited for this study. All participants were university students sharing a comparable level of education. Before recruitment, the participants received an e-mail detailing the experimental timeline, the inclusion and exclusion criteria, and the questionnaires. The Oldfield questionnaire (Oldfield, 1971) was used to determine handedness. The Pittsburgh Sleep Quality Index (PSQI) questionnaire (Buysse et al., 1989) was used to evaluate the sleep quality of the subjects. We also evaluated the subjective self-estimated hours of sleep per night. Finger dexterity was reported as the usual number of fingers of the non-dominant hand (left-hand for all participants) used when typing on a computer keyboard. Exclusion criteria were: left-handedness, neurological or psychiatric disorders (in particular, history of depression, epilepsy, brain injury, dizziness, and vertigo), severe sleep disorders (PSQI ≥8), medication or substances affecting the central nervous system, having undergone brain or vertebral column surgery, presence of medical devices (surgical clips, cochlear implants, drug pumps, pacemakers, et cetera), diagnosis of learning difficulties (dyslexia, dyscalculia, language impairments), tattoos or piercings on the scalp, professional level of typing or piano playing, pregnancy. Upon completion of all sessions, the participants received 8 € per hour as compensation. The data of four participants were excluded due to the automatic execution of a wrong sequence by the program during the task. Another six were excluded because they had consumed cannabis or alcohol, practiced the sequence or slept less than 4 h between the first and the second day of experiment. Thus, a total of 28 participants per group were included in the analysis.

The study conformed to the principles of the declaration of Helsinki and was approved by the local ethics committee of the Medical Faculty at the University of Würzburg. All subjects gave their written informed consent before the investigation. All participants were naive to the rationale of the experiment.

### Research Design

The participants were tested in a double-blind between-subject design as follows: (1) the group “Before” received cerebellar anodal tDCS (ca-tDCS) before the motor learning task; (2) the group “During_sham_” received sham tDCS during the task; (3) the group “During_real_” had real ca-tDCS during the task; and (4) the participants in the “After” group were stimulated with ca-tDCS after the task. The experiments were conducted on two consecutive days with 24 h between sessions. On the first day, subjects received tDCS and performed the motor learning task. On the second day, participants reported on their current health condition, their caffeine and drug consumption, their medication intake as well as the number of hours of sleep in the night between sessions. They subsequently performed the motor task. All experiments were conducted in the same room by the same investigator between 7 a.m. and 1:15 p.m.

### Finger Tapping Task (FTT)

**Figure 1:**
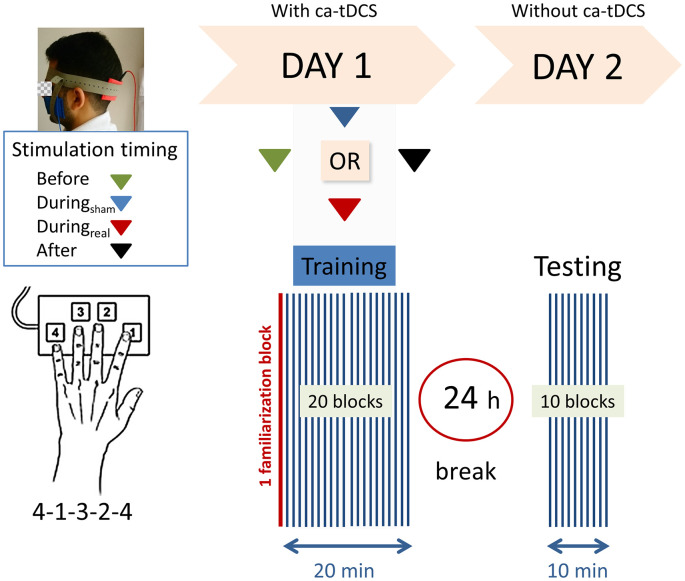
Experimental design. Right-handed participants were asked to perform as rapidly and accurately as possible a 5-digits sequence (4-1-3-2-4) of a finger-tapping task (FTT) on a modified keyboard using their left hand. One block consisted of 40 s of tapping and 20 s of rest. After a familiarization block (marked in red), the subjects performed 20 blocks on the first training day. Following a 24 h break, a recall session consisting of 10 blocks was completed. Anodal transcranial direct current stimulation was applied to the ipsilateral cerebellar hemisphere on day 1 with the reference electrode on the buccinator muscle at the following timings with respect to the training: (1) “Before,” (2) “During_sham_,” (3) “During_real_,” or (4) “After.”

Figure 1 provides a schematic view of the experiment. We used a computerized version of the sequential FTT initially developed by Karni et al. (1995). The study was designed using OpenSesame_3.1.4-wind32 (RRID:SCR_002849, Mathôt et al., 2012). Four keys, located in ergonomic positions on a computer keyboard were used (with keys-to-number assignment: 1 = index finger [N key], 2 = middle finger [G key], 3 = ring finger [D key], 4 = little finger [Y key]). The corresponding sequence was “Y-N-D-G-Y” on a keyboard with a standard QWERTZ layout (Fujitsu Technology Solutions). To limit the errors, white squared paper straps with the black written numbers (1, 2, 3, and 4) were taped over the keys, and all the letter keys that were not required for the task were removed from the keyboard. The task required to repeat, as quickly and accurately as possible, a sequence of five finger-movements (4-1-3-2-4) using the left, non-dominant hand for a period of 40 s followed by a break of 20 s. During the 20 s of the break, the message “20 s break” was displayed on the screen. A block lasted 1-min corresponding to 40 s of test and 20 s of the break. On the first day, participants were familiarized with the sequence during the one familiarization block before starting the real test. The familiarization trial did not differ from the other blocks and there was no notification between the familiarization trial and the beginning of the real session. The training session consisted of 20 blocks and lasted 20 min. All participants were instructed to concentrate on the task and not to talk. At the end of the session, participants were instructed not to practice the sequence outside of the experiment and to keep their usual sleep schedule between the 2 days of the experiment. On the second day, there was no initial familiarization trial and the subjects performed only 10 blocks of the same finger tapping sequence.

### Cerebellar Anodal tDCS (ca-tDCS)

Anodal cerebellar tDCS (DC-Stimulator Plus, neuroConn, Ilmenau, Germany, RRID:SCR_015520) was delivered through two 5 cm × 5 cm (25 cm^2^) electrodes soaked in a 0.9% NaCl-solution for at least 10 min. The anodal electrode was placed over the left cerebellar cortex, 3 cm lateral to the inion, and the cathodal electrode was placed on the left buccinator muscle (Galea et al., 2011). For the “During” groups, both the participants and the experimenter were blinded to the type of stimulation (sham or anodal) using the “study mode” implemented in the tDCS device. At the onset of stimulation, the current was increased in a ramp-like fashion for 10 s. For real stimulation, a 2 mA current (current density 0.08 A/cm^2^) was applied for 20 min. For sham stimulation, tDCS started with a short linear fade-in phase, followed by 2 mA of direct current for 30 s and a short linear fade-out phase. During the main time of the stimulation, only small current pulses occurred every 550 ms (110 μA over 15 ms) to check the impedance. The total stimulation session lasted 20 min, independently of the stimulation mode (real or sham). The timing of stimulation relative to the FTT (before, during, or after) varied according to the experimental group. All participants were instructed not to talk during stimulation. tDCS was well tolerated and there were no adverse effects.

### Data Analysis and Statistics

To evaluate FTT performance, we calculated the number of correct sequences per block on the first and second sessions for each participant. The change in performance throughout the training session on the first day was considered a measure of online learning. The online gains were calculated by subtracting the average performance in the first three blocks (PRE) from the average performance in the last three blocks (POST) on the first day of training:

(1) Online gains=POST−PRE

The difference between the average performance in the last three blocks of learning (POST) and the first three blocks of the recall session (RT) represented an index of off-line consolidation (Saucedo Marquez et al., 2013):

(2) Offline gains=RT−POST

SPSS version 22 (IBM, RRID:SCR_002865) was used for inferential analysis. The Shapiro–Wilk test was used to test normality and the Levene test was used to test homogeneity of variance. The Kruskal–Wallis one-way analysis of variance was used in case of non-normal distribution. We compared the normally distributed data using repeated-measures ANOVA with GROUP as between subjects’ factor (“Before,” “During_sham_,” “During_real_,” and “After”) and TIME as a within-subjects factor (T1 and T2). Online and offline gains for each group were compared using the one-way ANOVA test. The effect sizes were calculated as partial *η*^2^. Mauchly’s test was used to test the assumption of sphericity between the conditions and Greenhouse-Geisser correction was applied in case of violation. Bonferroni correction was applied for multiple comparisons. Data are reported as mean ± standard error of the mean. The significance level was set at *p* < 0.05.

## Results

### Demographic and Psychometric Data

Demographic and psychometric data are summarized in Table 1. Participants were between 19 and 27 years old, with no significant age differences between groups. All subjects were right hand dominant (handedness score 20–100%). There were no significant differences between the groups regarding sleep quality (PSQI) within the last 4 weeks before the experiment, self-estimated hours of sleep per night, and finger dexterity.

**Table 1:** Summary of demographic and psychometric parameters.

	Groups	*N*	Mean of ranks	*X*^2^	*p*
Age (years)	During _sham_	28	53.07		
	Before	28	57.30		
	During_real_	28	58.09	0.44	0.93
	After	28	57.54		
Handedness	During _sham_	28	51.61		
	Before	28	53.18		
	During_real_	28	59.54	1.94	0.59
	After	28	61.68		
Pittsburg Sleep Quality Index (PSQI)	During_sham_	28	52.45		
	Before	28	51.46		
	During_real_	28	61.70	2.3	0.51
	After	28	60.39		
Self-estimated sleep/night (hours)	During_sham_	28	56.61		
	Before	28	56.34	0.19	0.98
	During_real_	28	58.38		
	After	28	54.68		
Finger dexterity	During_sham_	28	51.89		
	Before	28	53.18	2.97	0.39
	During_real_	28	64.71		
	After	28	56.21		
Total of participants	*N* =	112			

### Effect of Cerebellar Anodal-tDCS on FTT Performance

**Figure 2:**
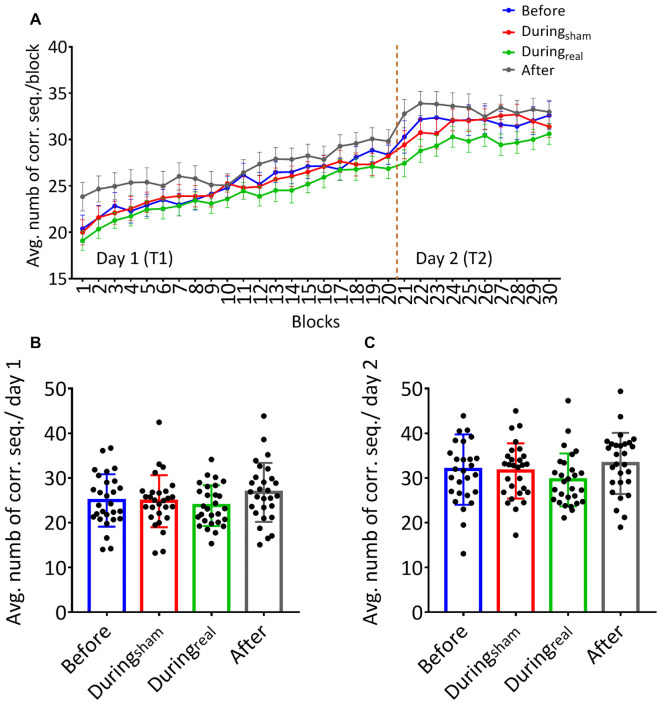
**(A)** Learning curves of the finger sequence tapping task. The number of correct sequences tapped during each block was collected and the average per block was calculated for each group on day 1 and day 2. Solid lines indicate group means, error bars indicate standard error of means (sem, *n* = 28 per group). **(B)** Mean performance on day 1. The overall average of correct sequences was calculated for the 20 blocks completed on day 1 for each group. **(C)** Mean performance on day 2. The overall average of correct sequences was calculated across the 10 blocks performed on day 2 for each group (mean performance ± SD, *n* = 28 per group).

Subjects received the tDCS stimulation at different timings (before, during, or after the task). No adverse effect was reported during or following the stimulation. On day 1, participants increased the number of correct sequences during the task as shown by the increasing learning curve across blocks (Figure 2A). We performed a two-way repeated-measures ANOVA with *GROUP* as between subjects’ factor and *TIME* as a within-subjects’ factor. There was no significant *GROUP × TIME* interaction on day 1 (*F*_(25.78,902.43)_ = 0.82, *p* = 0.72, *η*^2^ = 0.023) indicating that the average number of correct sequences per block was comparable between groups and that the timing of tDCS did not affect that performance. We found a main effect of time (*F*_(8.59,902.43)_ = 60.53, *p* < 0.001, *η*^2^ = 0.36) indicating significant online learning (practice effect) in every group.

Similarly, there was no significant *GROUP × TIME* interaction on day 2 (*F*_(12.74,445.74)_, *p* = 0.091, *η*^2^ = 0.043). Similarly to day 1, we found a significant main effect of time (*F*_(4.25,445.74)_ = 1.57, *p* = 0.03, *η*^2^ = 0.04) underlining that the average number of correct sequences performed per block further improved on the second day of FTT.

One-way ANOVA was used to compare the overall average of correct sequences for 20 blocks and 10 blocks on day 1 and day 2, respectively, in each group. We found no between-group differences on the overall average of correct sequences per group on day 1 (*F*_(0.96,52)_, *p* = 0.42, *η*^2^ = 0.12, Figure 2B). Similarly, there was no significant difference on the overall average of correct sequences per group on day 2 (*F*_(39.2,431)_, *p* = 0.91, *η*^2^ = 0.04, Figure 2C).

### Effects of the Timing of Cerebellar Anodal tDCS on Online and Offline Learning

**Figure 3:**
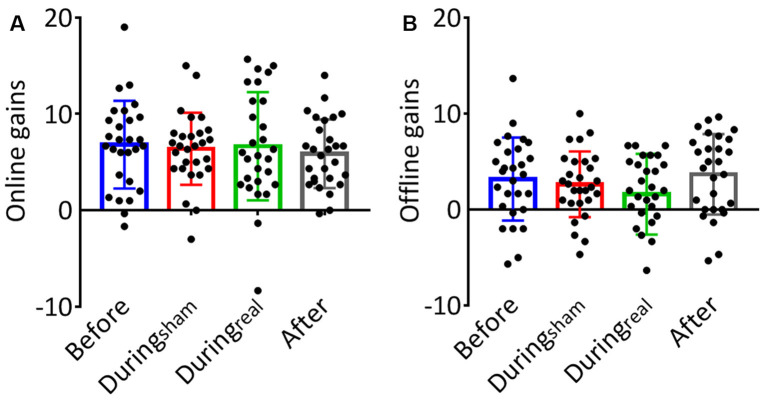
**(A)** Online gains. The change in performance throughout the training session on day 1 was considered a measure of online (or practice-dependent) learning. This gain was calculated by subtracting the average number of correct sequences in the first three blocks from the average number of correct sequences in the last three blocks on day 1. **(B)** Offline gains. The change in performance from the last three blocks of learning (day 1) to the first three blocks in the recall session (day 2) represented an index of offline gains. These gains were considered a measure of offline memory consolidation for each group (mean gain ± SD, *n* = 28 per group).

The results of the one-way ANOVA are shown in Table 2. We evaluated the online gain in the performance by subtracting the mean of correct sequences in the first three blocks from the mean of correct sequences in the last three blocks for each group and day. There was no significant difference between groups at day 1 (*p* = 0.59, Figure 3A). To evaluate the consolidation/retention effect, we subtracted the average of the correct sequences in the first three blocks of the second day from the last three blocks in the first day. Again, there was no significant difference between groups (*p* = 0.27, Figure 3B).

**Table 2:** Online and offline gains in the finger-tapping task (FTT).

		*N*	Mean	Standard deviation	*F*	Partial *η*^2^	*p*
Online	During_sham_	28	6.38	3.73			
gains	Before	28	6.82	4.55			
day 1	During_real_	28	6.65	5.61	0.65	0.01	0.59
	After	28	5.32	3.47			
Offline	During_sham_	28	2.64	3.42			
gains	Before	28	3.18	4.32			
	During_real_	28	1.61	4.21	1.34	0.03	0.27
	After	28	3.68	4.19			
Total of participants	*N* =	112					

## Discussion

We evaluated the impact of timing of ca-tDCS applied before, during, or after an FTT (relative to sham) on the online motor performance and task retention. We found that all participants showed an improvement in performance as measured by the number of correct FTT sequences for each session. There was no effect of cerebellar tDCS on motor learning. Participants who received sham stimulation produced the same average of correct responses as participants who received active anodal tDCS either before, during, or after the task. There were no baseline differences in motor performance at the beginning of the task on day 1. Similarly, there was no difference in online gains or offline improvement between groups. Overall, our results underline a lack of effect of ca-tDCS on motor learning in the context of FTT.

### Cerebellar tDCS Did Not Affect Online Learning

In the context of this study, it is important to discriminate between motor adaptation and skill learning. While in motor adaptation, the learner adapts to the error induced by a perturbation and shows an aftereffect when the perturbation is removed (Martin et al., 1996), motor skill learning is evaluated through exposure to a novel motor task. Successful learning is measured through the reduction of errors and performance improvement beyond baseline levels (Reis et al., 2009). To our knowledge, our study is the first to evaluate the impact of timing of a single ca-tDCS on the performance in an FTT, a motor sequence learning task that has been commonly associated with cerebellar activation (Witt et al., 2008). While cerebellar activity has traditionally been associated with externally cued movements, it is also observed in motor tasks driven by internal cues (Grafton et al., 1992). Specifically, lobule VIII of the cerebellum is recruited during out-of-phase movements related to the motor coordination of finger movements (Habas et al., 2004). Moreover, right-handed finger-tapping activated right cerebellar lobules IV-V and VIII (Stoodley et al., 2012). In a recent modeling study, the electrode montage used in our current study has consistently been demonstrated to generate an electrical field with its peak strength within cerebellar lobules VII and VIII (Moussa-Tooks et al., 2020).

The participants in this study did not show baseline differences in task performance. Average age, sleep quality, handedness, and finger dexterity did not differ between the four groups. We evaluated and compared the change in motor performance during the task as a measure of online gains and found no differences between the groups. Our result underlines a lack of effect which is in contradiction with some previous studies of others showing a positive effect of ca-tDCS on motor learning in various tasks. For example, ca-tDCS enhanced motor performance by improving the rate of adaptation when compared to sham stimulation (Jayaram et al., 2011). In paradigms that specifically involve the upper limb, ca-tDCS improved the accuracy index in a skilled angle tracking task (Shah et al., 2013) and reduced the response time and the number of errors in the serial response time task (Ehsani et al., 2016; Samaei et al., 2017). Controversially, other authors reported a lack of effects of the anodal stimulation in a specific upper limb test with an adaptation component like the visuomotor adaptation task (Panouillères et al., 2015; Liew et al., 2018). Moreover, a study from Taubert et al. (2016) suggested that ca-tDCS impaired motor memory acquisition and increased motor errors during the re-acquisition of an original motor memory in a force field perturbation-reaching task.

In most of the above-mentioned studies, the current was delivered during the task, ipsilateral to the trained limb at a current density of 0.08 mA/cm^2^. This condition is comparable to the “During_real_” group in our study where participants received the stimulation during the task. The “During_real_” group showed no significant online gains compared to the “During_sham_” group that received sham stimulation. We also evaluated the online gains when the active stimulation was delivered before or after the task and found similar results, which confirmed the lack of effect of ca-tDCS.

It is however important to mention that we specifically stimulated the left cerebellum, whereas most of the up-to-date studies published have stimulated the right cerebellum. This difference in the hemisphere stimulation might influence the cortical response (Schlerf et al., 2015) indicating some laterality differences in the cerebellar-motor cortex connectivity. As such, they observed in right-handed individuals that the connection between the right cerebellum and left M1 was stronger than the contralateral network. This possibility of lateralization might have influenced the results in our right-handed participants.

### Cerebellar tDCS Did Not Affect Task Retention (Offline Gains)

Motor performance in the FTT was re-assessed after a break of 24 h as a measure of task retention and offline learning. Our results indicated no impact of tDCS on task retention. Similarly as discussed for the online gains, reports about the effects of cerebellar anodal tDCS on offline gains are controversial. For instance, Taubert et al. (2016) reported an impaired early adaptation in a force field perturbation task at 24 h post-tDCS. This negative effect was also found as a decrease of reaction time in a serial reaction time task following anodal cerebellar stimulation. However, two other studies have reported a positive outcome on offline learning following ca-tDCS in the above-mentioned serial reaction task. This was reflected by a greater reduction in response time, but not the number of errors, in individuals over 40 years (Samaei et al., 2017) and a greater reduction in the number of errors and faster response time in subjects younger than 40 years old (Ehsani et al., 2016). In all those studies the stimulation was applied during the task in a single session. Similar to the result found with the “Before” group of our study, Foerster et al. (2017) have reported the absence of effect of ca-tDCS on offline learning in an adaptive balance control task when the stimulation was applied before the task. In this study, we have also tested a condition where anodal stimulation was applied after the task (“After” group) as another possible offline stimulation design. We found no difference between the groups that received the tDCS before or after completing the task on day 2. This result shows that tDCS after the task did not affect retention. From the literature, it is unclear whether cerebellar tDCS has any effect on motor learning, performance, and retention when applied during or after the task. Our current results point toward a lack of effect in both timings.

### Inconsistency in tDCS Studies and Limitations

Recent studies have called attention to several aspects of the methodology for tDCS (Thair et al., 2017). One of them includes the variability in performance level that was not assessed in this study. tDCS effects could have been masked by large inter-individual variability in motor skills. Another important factor is the hair thickness and texture that can increase impedance and thus allow less current to reach brain tissue (Doyon and Benali, 2005). The impedance in this study was maintained below 15 KΩ throughout the stimulation session. However, we did neither protocol individual impedance in detail nor did we map patients’ skulls or brain tissue and thus, we do not know whether groups differed in these matters significantly.

A potential contributor to the null effect might be the specificity of the electrode positioning that has been shown effective to facilitate motor adaptation (Morton and Bastian, 2006; Jayaram et al., 2012; Poortvliet et al., 2018). Ability to adapt to an external output and ability to learn a sequence of the movement have been often treated as distinct forms of motor learning. Both forms of motor learning might be supported by distinct neural substrates. Thus, work by Imamizu et al. (2000) has consistently shown brain activation in the cerebellar regions surrounding the posterior superior fissure of the cerebellum during adaptation of movements to differing visual distortions. On the other hand, cortico-cerebellar anatomical systems are crucial for mediating the acquisition and the execution of motor skills (Hikosaka et al., 2002; Doyon and Benali, 2005). These aspects of learning are not necessarily dissociated from real behaviors. Therefore, we argue that the stimulation of the posterior part of the cerebellum could modify performance in skill learning as underlined by the work of others (Wessel et al., 2016). However, it is now imperative to consider the use of additional techniques to the protocol that may map the cerebellum and its surrounding skull as well as the functional areas being investigated. Such techniques include the use of computational models, MEP or TMS (Furuya et al., 2014; Fan et al., 2017). Cerebellar Lobules Optimal Stimulation (CLOS) is an example of an open-source computational modeling pipeline that could support a critical and rational design as well as optimization of neuromodulation (Rauscher et al., 2020). The implementation of those tools represents an important change of the practice in the field that will improve the reproducibility of the studies.

The blinding methods have also been questioned in recent studies. The design of sham stimulation was conducted similarly to other studies (e.g., Jayaram et al., 2012; Manto et al., 2013; Poortvliet et al., 2018). Gandiga et al. (2006) have suggested that good blinding effects are achieved when using a ramp-like fade-in phase of approximately 10 s and turning off the current after 30 s in sham stimulation in comparison to a real stimulation applied at 1 mA for 20 min. These findings have been implemented by multiple tDCS studies presuming good blinding with similar protocols (Fregni et al., 2005, 2006). Based on the evaluation that we performed in half of our participants, only two subjects that received the sham stimulation were able to appropriately identify it. This means that most of the sham-stimulated participants thought that they have received an active stimulation. This result has been added to the Supplementary Material. However, we aim to monitor and assess the influence of the subjective experience of sensations during the stimulation as well as subjective allocation to either sham or anodal group to control insufficient blinding in the future.

The population in the present study was young, healthy, and well-educated. Participants performed already with very high accuracy in the first block and could therefore not improve much further during the experiments. Indeed, Furuya et al. (2014) compared the effects of tDCS on the fine control of sequential finger movements in highly trained pianists and musically untrained individuals. They demonstrated an improvement of fine motor control in both hands in musically untrained controls, but the deterioration in pianists following anodal tDCS over the contralateral cortex. This underlines that the effects of tDCS might be expertise-dependent. In the current study, participants were naïve to the task. We also purposely excluded subjects with extended typing experience, including piano playing. We can therefore assume that the subjects had a similar level of initial performance. This was also confirmed by the absence of difference in correct response in the first block of T1. We could speculate the real statistical effect hidden by the very high initial performance would be uncovered in older participants or participants with cerebellar functional impairment. However, a recent study has also shown a lack of cerebellar tDCS effect on learning of a complex whole dynamic balance test in middle-age adults (Rauscher et al., 2020) underlining that parameters beyond the age of participants are responsible for the null effect obtained in our study.

It is important to note that the results presented here and their interpretation are limited to the effect of a single stimulation. Possibly, multiple sessions could have yielded a different result. Anodal transcranial stimulation of the primary motor cortex over five consecutive days has been shown to enhance motor performance in a grip task in healthy young subjects (Fan et al., 2017). Cerebellar anodal tDCS over 3 days improved the online performance of healthy participants in a sequential visual isometric punch task (Cantarero et al., 2015). Therefore, we cannot exclude that with only one tDCS session, the effects may have been minimal, whereas multiple sessions could have shown larger and longer-lasting effects. However, there is no clear evidence to strongly support the effectiveness of multiple sessions over a single session when it comes to cerebellar stimulation. A recent systematic review summarizing the effects of cerebellar tDCS on motor learning has concluded to a potential positive effect of a single session of ca-tDCS in improving short to long-term motor skill learning beyond the training period (Kumari et al., 2019).

Finally, we cannot exclude that the effect of cerebellar at-DCS might have been missed because of the sample size (*N* = 28 per group). However, based on previous comparable studies performed with a similar number of subjects per group, we had performed an *a-priori* power analysis and had found that 49 subjects in total would be required to achieve a power of 0.8. A total of 112 participants were included in the current study, yet yielding a lack of effect. It is therefore possible that tDCS studies and especially cerebellar tDCS studies require substantially larger sample sizes because of the very small effects. To overcome the recruitment limitation, the pooling of data from several studies with small samples but similar experimental designs will create large data sets that might allow the estimation of efficacy much more precisely (Minarik et al., 2016).

## Conclusion and Implications

Cerebellar anodal t-DCS neither facilitated learning, nor retention in a FTT in young and healthy subjects. Several reasons explaining these results have been raised above. Our results call for a careful design of experiments using tDCS and underline the need for further investigation regarding the timing of its application, electrode placement, and intensity of the current as well as the choice of the target population in neurological studies. The individual differences, the sensitivity to tDCS, and the inter-population variability are among the parameters that could also play a critical role in the outcome of tDCS applications. It is becoming evident that heterogeneous and limited effects of tDCS including ours are calling for an urgent need to develop biomarkers that can help to predict individual response to non-invasive stimulation. We also need to define personalized targets and optimal parameters for the success of the intervention before moving to the clinical context where robust and consistent effects are expected despite the high variability of the subjects’ condition.

## Data Availability Statement

The original contributions presented in the study are included in the article/Supplementary Material, further inquiries can be directed to the corresponding author.

## Ethics Statement

The studies involving human participants were reviewed and approved by the ethics commission of the university hospital of Würzburg, Germany. The patients/participants provided their written informed consent to participate in this study.

## Author Contributions

CN: conceptualization, investigation, data acquisition, formal analysis, writing—original draft, writing—review and editing. AS and SH: recruitment, investigation, data acquisition, and formal analysis. DZ: conceptualization, writing—review and editing, funding acquisition, project administration, resources, and supervision. All authors contributed to the article and approved the submitted version.

## Conflict of Interest

The authors declare that the research was conducted in the absence of any commercial or financial relationships that could be construed as a potential conflict of interest.
