# Supplementary material for: No Impact of Cerebellar Anodal Transcranial Direct Current Stimulation at Three Different Timings on Motor Learning in a Sequential Finger-Tapping Task
Source: Front Hum Neurosci. 2021 Feb 5;15:631517. doi: 10.3389/fnhum.2021.631517 (PMC7892471; doi:10.3389/fnhum.2021.631517)
Supplement: Supplementary file 1 [file Table_1.DOCX]

Supplementary Table 1. Means, standard deviations, χ^2^-values, partial eta squared and p-values for the Kruskal Wallis H-test of the intensity of perceived sensations for each group. A total of 56 participants in this study completed a comfort rating questionnaire after the stimulation sessions. The intensity of 7 sensations (itching, pain, burning, warmth, metallic taste, fatigue, and other) were evaluated on a scale of 0-3 (None=0, Mild=1, Moderate=2, Strong=3). A Kruskal Wallis H-test showed a significant difference in the intensity of perceived sensations between the groups (p = .033).

|  |  | N | Mean | Std. Deviation | χ^2^ | Partial η^2^ | *p* |
| --- | --- | --- | --- | --- | --- | --- | --- |
| Intensity of | Before | 14 | 2.43 | 1.56 |  |  |  |
| Perceived | After | 14 | 2.50 | 2.10 |  |  |  |
| Sensations | During_real_ | 14 | 1.86 | 1.23 | 8.75 | 0.14 | .033 |
|  | During_sham_ | 14 | 1.00 | 1.18 |  |  |  |
| Total participants | N = | 56 |  |  |  |  |  |
