# Supplementary material for: No Impact of Cerebellar Anodal Transcranial Direct Current Stimulation at Three Different Timings on Motor Learning in a Sequential Finger-Tapping Task
Source: Front Hum Neurosci. 2021 Feb 5;15:631517. doi: 10.3389/fnhum.2021.631517 (PMC7892471; doi:10.3389/fnhum.2021.631517)
Supplement: Supplementary file 2 [file Table_2.DOCX]

Supplementary Figures

*Supplementary Figure 1A.* The mean intensity of perceived sensations reported by the participants in each of the four groups. A total of 56 participants in this study completed a comfort rating questionnaire after the stimulation sessions. The intensity of 7 sensations (itching, pain, burning, warmth, metallic taste, fatigue, and other) were evaluated on a scale of 0-3 (None=0, Mild=1, Moderate=2, Strong=3). A Kruskal Wallis H-test showed a significant difference in the intensity of perceived sensations between the groups (*p* = 0.033). Despite this difference, the participants showed only a mild discomfort during the tDCS sessions as the average intensity was <3 (out of a maximum score of 21) in all the groups.

*Supplementary Figure 1B.* The number of participants in each group that correctly guessed their stimulation condition. Participants made their responses in the comfort rating questionnaire administered after the stimulation session. They responded either “Real”, “Placebo” or “I don’t know” when asked to guess their stimulation condition. Only 2 participants in the sham group correctly guessed that they were receiving sham stimulation. This result indicated an effectiveness of blinding. *(Ntotal = 56; Ngroup=14)*
