# Supplementary figures and images for: No Impact of Cerebellar Anodal Transcranial Direct Current Stimulation at Three Different Timings on Motor Learning in a Sequential Finger-Tapping Task
Source: Front Hum Neurosci. 2021 Feb 5;15:631517. doi: 10.3389/fnhum.2021.631517 (PMC7892471; doi:10.3389/fnhum.2021.631517)

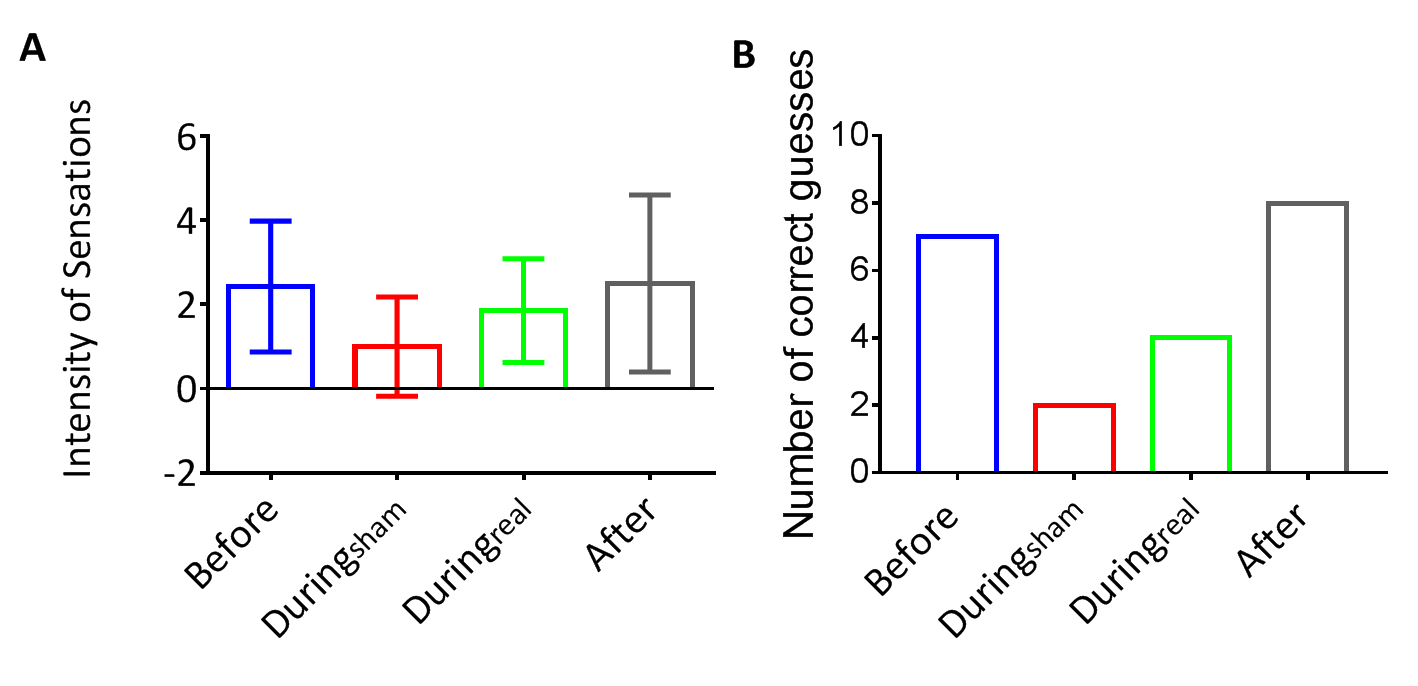

Supplement: Supplementary file 3 [file Image_1.TIF]
